# Allele-specific expression in the human heart and its application to postoperative atrial fibrillation and myocardial ischemia

**DOI:** 10.1186/s13073-016-0381-1

**Published:** 2016-12-06

**Authors:** Martin I. Sigurdsson, Louis Saddic, Mahyar Heydarpour, Tzuu-Wang Chang, Prem Shekar, Sary Aranki, Gregory S. Couper, Stanton K. Shernan, Jon G. Seidman, Simon C. Body, Jochen D. Muehlschlegel

**Affiliations:** 1 Department of Anesthesiology, Perioperative and Pain Medicine, Brigham and Women’s Hospital / Harvard Medical School, 75 Francis Street, Boston, MA 02115 USA; 2 Division of Cardiac Surgery, Department of Surgery, Brigham and Women’s Hospital / Harvard Medical School, Boston, MA USA; 3 Division of Cardiac Surgery, Department of Surgery, Tufts Medical Center, Boston, MA USA; 4 Department of Genetics, Harvard Medical School, Boston, MA USA

## Abstract

**Background:**

Allele-specific expression (ASE) is differential expression of each of the two chromosomal alleles of an autosomal gene. We assessed ASE patterns in the human left atrium (LA, n = 62) and paired samples from the left ventricle (LV, n = 76) before and after ischemia, and tested the utility of differential ASE to identify genes associated with postoperative atrial fibrillation (poAF) and myocardial ischemia.

**Methods:**

Following genotyping from whole blood and whole-genome sequencing of LA and LV samples, we called ASE using sequences overlapping heterozygous SNPs using rigorous quality control to minimize false ASE calling. ASE patterns were compared between cardiac chambers and with a validation cohort from cadaveric tissue. ASE patterns in the LA were compared between patients who had poAF and those who did not. Changes in ASE in the LV were compared between paired baseline and post-ischemia samples.

**Results:**

ASE was found for 3404 (5.1%) and 8642 (4.0%) of SNPs analyzed in the LA and LV, respectively. Out of 6157 SNPs with ASE analyzed in both chambers, 2078 had evidence of ASE in both LA and LV (*p* < 0.0001). The SNP with the greatest ASE difference in the LA of patients with and without postoperative atrial fibrillation was within the gelsolin (*GSN*) gene, previously associated with atrial fibrillation in mice. The genes with differential ASE in poAF were enriched for myocardial structure genes, indicating the importance of atrial remodeling in the pathophysiology of AF. The greatest change in ASE between paired post-ischemic and baseline samples of the LV was in the zinc finger and homeodomain protein 2 (*ZHX2*) gene, a modulator of plasma lipids. Genes with differential ASE in ischemia were enriched in the ubiquitin ligase complex pathway associated with the ischemia-reperfusion response.

**Conclusions:**

Our results establish a pattern of ASE in the human heart, with a high degree of shared ASE between cardiac chambers as well as chamber-specific ASE. Furthermore, ASE analysis can be used to identify novel genes associated with (poAF) and myocardial ischemia.

**Electronic supplementary material:**

The online version of this article (doi:10.1186/s13073-016-0381-1) contains supplementary material, which is available to authorized users.

## Background

Genetic variation causes disease through alteration in the quantity and function of proteins, among other mechanisms [1]. Expression of a gene’s messenger RNA (mRNA) is controlled by numerous mechanisms that are influenced by local and distant genetic variation, such as single nucleotide polymorphisms (SNPs).

Quantifying gene expression by RNA sequencing (RNA-seq) is performed by alignment of short RNA sequence reads from a tissue that is mapped to a reference genome sequence. The number of measured mRNA reads accurately measures gene expression [2]. Methods utilizing high-throughput RNA-seq have been used to study the genetic background of multiple cardiovascular diseases. This has thus far mostly been performed in animal models, exemplified by the assessment of changes in gene expression in mouse models of myocardial ischemia [3, 4]. In humans, we recently used this technique to measure the effects of ischemia on the gene expression of the left ventricle (LV) during cardiopulmonary bypass [5]. This indicated substantial changes in the expression of a wide variety of functional categories of genes between the baseline and post-ischemic samples in a short amount of time.

Differences in expression between each of the two SNP alleles in autosomal genes, called allele-specific expression (ASE) [6], allows us to examine the biological control of specific gene expression in health and disease [7]. This technique has successfully quantified ASE across a host of tissues, revealing that 2.3% of all tested SNPs control nearby gene expression, with substantial tissue-specific variation and moderate similarity in the ASE pattern of similar tissue types [8].

Studies on ASE in specific organs are sparse, especially using living tissues, and the ASE landscape of the human heart is poorly understood. Furthermore, limited data exist to show that ASE analysis can be useful to understand the pathophysiology of cardiac disease. Here we utilized two unique high-throughput RNA-seq datasets from the human left atrium (LA) and left ventricle (LV) during open heart surgery. We used these data to discover ASE in these tissues after filtering and quality control of both SNPs and RNA reads to call ASE. Our hypothesis was that there was ASE common to both chambers, as well as chamber-specific ASE. Furthermore, we assessed whether ASE could be used to identify novel variants associated with cardiac disease by comparing differential ASE between patients who had postoperative atrial fibrillation (poAF) and those who did not, and to find variants with differential ASE associated with the myocardial response to ischemia.

## Methods

### Patient cohorts

We obtained tissue samples from two prospective studies utilizing next-generation RNA-seq and high-density genome-wide DNA genotyping. Samples from the LA came from a cohort of 62 Caucasian patients undergoing mitral valve repair or replacement surgery for mitral valve regurgitation with cardiopulmonary bypass. During incision of the LA to access the mitral valve, a small sample of the left atrial free wall was obtained and used for RNA isolation.

LV samples were obtained during placement of the LV vent in 76 Caucasian patients undergoing aortic valve replacement for aortic stenosis with or without concomitant coronary artery bypass grafting. A small punch biopsy of the anterior apex of the LV was obtained at two time points: immediately after aortic cross clamping (baseline) and shortly before aortic cross clamp removal (post-ischemia) as described previously [5]. Between samples, cold blood cardioplegia was intermittently administered to reduce the extent of myocardial ischemia. There was no patient overlap between the LA cohort and LV cohort.

Patient demographics, surgical, and clinical outcome phenotypes were collected prospectively. Patients who donated LA samples were followed for poAF, defined as AF identified by any clinician during the primary hospitalization.

### RNA-seq and genotyping

For both studies, tissue samples were placed in RNAlater (Ambion; ThermoFisher Scientific) solution, followed by whole RNA extraction using standardized methods [5]. After reverse transcription of single-stranded RNA to double-stranded DNA, isolation of short fragments, poly(A) addition and ligation of adaptors, double-stranded sequencing was performed on an Illumina HiSeq 2000 (Illumina, San Diego, CA, USA). Read length for the samples was in the range of 90–100 bp.

DNA was isolated from whole blood using standard methods. SNP genotyping was performed using the Illumina Omni2.5 array with additional exome content (Illumina, San Diego, CA, USA) chip, version 1.1 for the LV samples and version 1.2 for the LA samples.

### Sequence alignment and processing for ASE calling

After RNA-seq, adaptor sequences and low-quality reads were removed, TopHat2 and BowTie algorithms were used to align the sequenced reads to the human genome (hg19) [9]. A list of heterozygous SNPs for each individual was generated and the RNA sequences that uniquely overlapped the location of each heterozygous SNP were identified [10]. From the patient mRNA sequence reads and the location of heterozygous SNPs in each patient, the appropriate base read at the location of each heterozygous individual was extracted and counted using SAMtools and custom made scripts [10].

### ASE calling and statistics

All statistics and imaging was done in R, version 3.1.0. Several filters were applied to identify SNPs appropriate for ASE calling [11]. Only SNPs with at least one heterozygous individual were analyzed. A minimum of 15 RNA reads crossing the heterozygous SNP were required. SNPs were also required to be in Hardy–Weinberg equilibrium (*p* > 0.00001) and have a genotyping rate of more than 95%, to be included. Only SNPs in regions with a mappability score of 1 were included, indicating good mapping of short reads to the region. Only reads with a unique mapping to the genome were used. Finally, we estimated the overall genotyping error (ratio of the counts of alleles other than reference and alternative allele to the overall number of allele counts). Using this estimate, we tested the null hypothesis that the number of alleles other than the reference and alternative alleles was the same as the overall genotyping error. The SNPs where the null hypothesis was rejected at *p* < 0.05 were considered to potentially have evidence of genotyping error or random monoallelic expression and were excluded from further analysis.

After the application of each filter, we assessed the reference sequence ratio, defined as REF/ALT + REF, where REF is the read count of the allele listed in the human reference genome (hg19) and ALT is the read count of alternative genome allele. An overall ratio of 0.5 indicates an equal number of reads of reference and alternative allele, indicating no preference for the allele listed in the reference genome used for aligning (reference genome bias). This can be used for individual SNPs to assess ASE and also as a summary statistic over all SNPs to assess reference genome bias.

We evaluated both the traditional binomial test and algorithms utilizing different statistical distribution to call ASE, with the goal of reducing the number of false positives and reference genome bias. A binomial test was used for each SNP using the sum of REF and ALT allele counts over all individuals. This tests the REF/ALT + REF ratio against the null hypothesis of a REF/ALT + REF ratio of 0.5. The *edgeR* package was used to call ASE, utilizing negative binominal distribution and estimation of individual sample and variant expression dispersion [12]. This was performed using both the sum of REF and ALT allele counts with a fixed dispersion estimate of 0.1 and also by using REF and ALT allele counts from each individual. Alternatively, the *limma* package was used to call ASE after *voom* transformation of the count matrix using REF and ALT allele counts from each individual [13]. The results of the algorithms were compared by QQ and Venn plots to visualize the number of SNPs/genes with ASE (Additional file 1: Figure S1-S3). The ASE calling was assessed visually by plotting the REF/ALT + REF ratio versus *p* value of the ASE assumption (Additional file 1: Figure S4). A false discovery rate (FDR)-adjusted *p* value < 0.05 was used to avoid overcalling ASE, and considered indicative of ASE.

After ASE calling in the LA and LV samples, SNPs with ASE in both chambers were identified, as well as SNPs with chamber-specific ASE in either LA or LV but not the other. The absolute number of shared SNPs was compared against the distribution of the number of shared SNPs with 10,000 permutations of a random sample of eligible SNPs.

The left atrial expression of reference and alternative alleles of each heterozygous SNP was compared between patients who did and did not have poAF. Differential ASE during ischemia was tested by comparing the expression of the reference and alternative alleles of each heterozygous SNP between the baseline and the post-ischemia sample, using paired analysis of the LV samples. A functional enrichment analysis was performed on the gene sets with differential ASE (at FDR-adjusted *p* value <0.05) using the GeneMANIA algorithm within the Cytoscape network visualization tool [14, 15]. This analysis was performed using the default interaction networks with the exception of the default co-expression networks, which were exchanged for custom-made LA and LV co-expression networks using our gene expression data. The algorithm allowed the inclusion of the top 20 related genes and at most 20 attributes using automatic weighting.

### Validation cohort

To contrast our result against an independent set of data, we downloaded the ASE dataset from the Genotype-Tissue Expression (GTEx) pilot analysis. The generation of this dataset has been described in detail elsewhere [8]. In short, the dataset contains results from RNA-seq, both exome sequencing and genome-wide RNA-seq of various tissues in several hundred deceased individuals after variable amount of warm ischemic time. Sequence alignment and quality control of genotyping is similar to the one done in this study. The GTEx dataset release contains counts of reference and alternative alleles of heterozygous SNPs. We extracted from this dataset counts of reads overlapping reference and alternative alleles of heterozygous SNPs from the left atrial appendage tissue and from the left ventricular tissue. After filtering the available SNPs using the same minimum number of overlapping reads and both mappability and read counts, we applied the same filters of minimum read numbers and mappability criteria, and then called ASE with the edgeR algorithm based on individual allele counts. For those SNPs available for ASE analysis in both our LA tissue and the GTEx left atrial appendage tissue, we compared the number of SNPs with ASE in both datasets with the number of ASE in either dataset. This was contrasted with the same statistic after 10,000 random permutations of the eligible SNPs. The same analysis was performed for SNPs in our LV tissue set and the GTEx LV tissue.

## Results

### Patient demographics

The mean age of patients who had LA sampling (n = 62) was 58 years and 44% were female. Following the surgery, 21 (34%) patients had poAF, defined as any atrial fibrillation diagnosed by clinician during the initial postoperative hospitalization. The patients with poAF were older (65 versus 56 years, *p* = 0.006) and had a higher rate of hypertension (81% versus 41%, *p* = 0.01), but the groups were otherwise highly similar. There was no difference in the past history of myocardial infarction, diabetes, or previous history of atrial fibrillation. Similarly the rates of preoperative use of renin-angiotensin-aldosterone inhibitors, beta-blockers, calcium channel blockers, and statins were similar between the two groups. Furthermore, although LA volume was enlarged (mean LA volume 71 mL/m^2^), there was no difference in LA volume between the two groups. Similarly, cardiopulmonary bypass and aortic cross-clamp time was comparable between the two groups (data not shown).

The mean age of patients who had paired LV sampling (n = 76) was 74 years and 42% were female. The vast majority (86%) of patients had LV ejection fraction of more than 50%. A total of 43 patients (56%) had concomitant coronary bypass surgery alongside the aortic valve replacement. The median ischemic interval created by aortic cross-clamping was 90 min (range 48–284).

### Filtering of RNA reads and SNPs and selection of ASE calling algorithm

After removal of low-quality reads, trimming of adaptor sequences, and alignment to the human genome, a total of 421,780,889 reads from the LA samples overlapped a heterozygous SNP in at least one individual. Of those, 9,451,851 reads were not uniquely mapped to the human genome and were removed, leaving 412,329,038 reads for analysis. Similarly, from the LV samples a total of 1,879,293,644 reads overlapped a heterozygous SNP in at least one individual. Of those, 158,775,428 reads were not uniquely mapped, leaving 1,720,518,216 reads for analysis.

After filtering SNPs with an inadequate number of overlapping reads and those failing Hardy–Weinberg equilibrium, along with cross-checking for minimum genotyping rate and mappability criteria, a total of 112,020 and 214,626 SNPs were available from the LA and LV samples, respectively (Additional file 1: Table S1). There was a substantial improvement in reference genome bias following SNP filtering (Additional file 1: Figure S1).

There was a high agreement of ASE calling between binomial test and ASE called by both the *edgeR* and *limma* algorithms. However, there was a substantial variability in the absolute number of SNPs with significant ASE (Additional file 1: Figure S2). The *edgeR* ASE calling using individual sampling, which was used for further analysis, was sufficiently conservative in both the LA and LV samples and the QQ curves had a similar shape for both tissues (Additional file 1: Figure S3). From the SNPs with ASE based on this algorithm (FDR-adjusted *p* < 0.05), a higher number of SNPs had REF/ALT + REF ratio greater than 0.5 than a ratio less than 0.5 in both samples (3383 (59%) versus 2305 (41%), *p* < 0.001 for the LA samples, and 5539 (64%) versus 3103 (36%), *p* < 0.001 for the LV samples) (Additional file 1: Figure S4). This indicates some residual reference genome bias in the SNPs with ASE called. We furthermore observed that this bias was more prominent among SNPs with fewer covering reads (Additional file 1: Figure S5).

### SNPs and genes with ASE in LA and LV

**Fig. 1:**
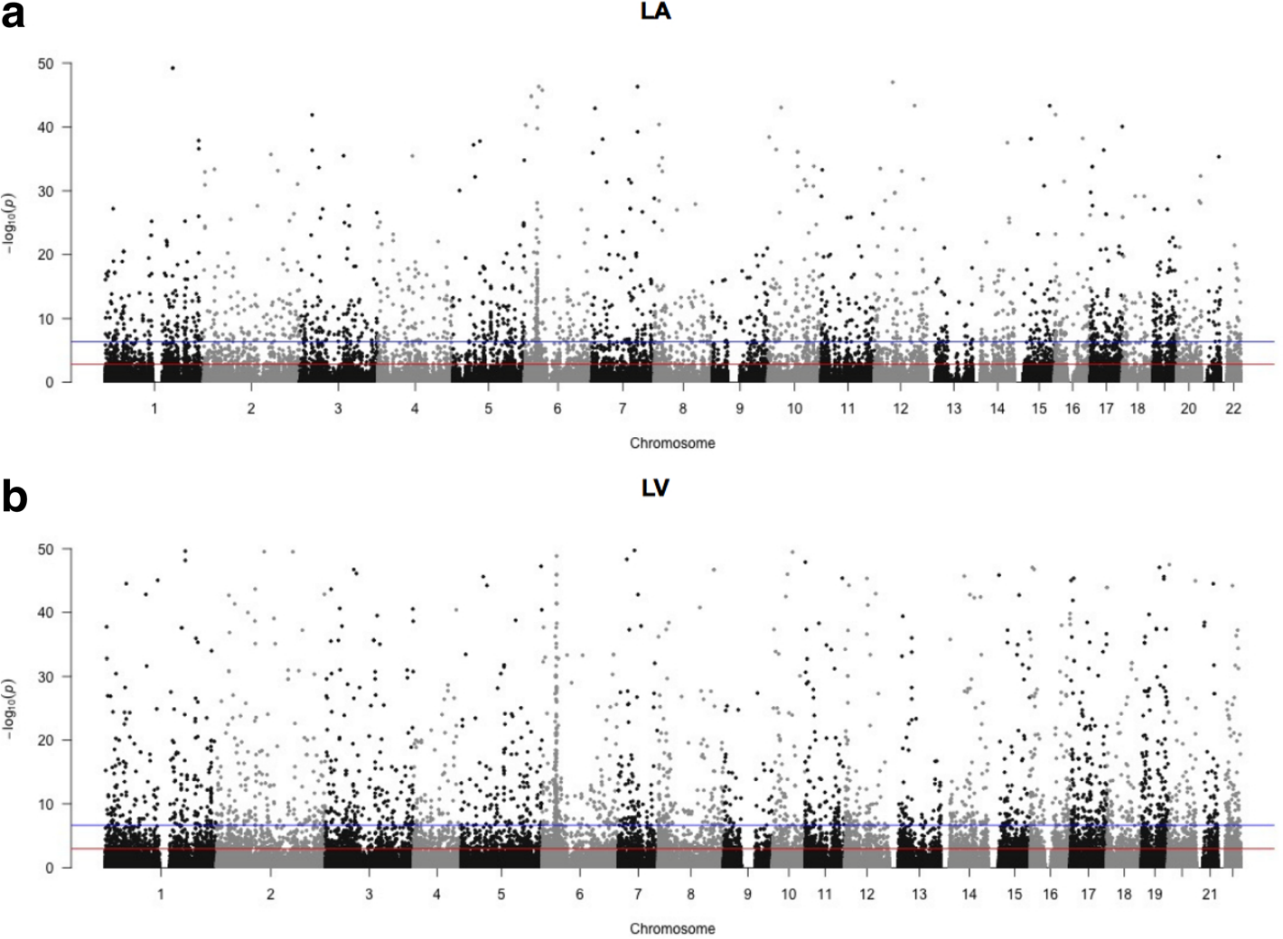
A *Manhattan plot* showing the distribution of SNPs with ASE in (**a**) left atrium (LA) and (**b**) left ventricle (LV). Horizontal lines indicate *p* = 0.05 (*solid red*) and a Bonferroni-adjusted *p* value of 0.05/n where n is the number of SNPs tested in each tissue (*blue*)

In the LA samples, there were 12,768 SNPs with significant ASE at *p* < 0.05 (Additional file 2). Of those, 5688 had an FDR-adjusted *p* value < 0.05 (Fig. 1a). Table 1 shows the ten SNPs with the most significant ASE and Fig. 1a shows the genomic distribution of all SNPs with ASE in the LA. From 55,984 SNPs available for analysis in our LA samples and the genome-wide LA appendage samples from GTEx, 1356 had evidence of ASE in both sample sets (*p* < 0.0001 by permutation, Additional file 1: Figure S4). Similarly, of 24,830 SNPs available for analysis in our LA samples and the exome-sequencing LA appendage samples, 688 had evidence of ASE in both sample sets (*p* < 0.0001 by permutation, Additional file 1: Figure S7).

**Table 1:** SNPs with allele-specific expression in the LA

SNP	Gene	Chr	Location	Samples	Reads	REF/ALT + REF	FDR-adj. *p* value
rs3810232	*RPS9*	19	54,704,760	35	33,337	0.05	<10^–300^
rs111794229	*TIMP3*	22	33,257,378	23	9569	0.89	<10^–300^
rs202118461	*LYRM5*	12	25,357,576	32	3253	0.86	<10^–300^
rs112332443	*TGOLN2*	2	85,545,951	15	2395	0.95	2 × 10^–293^
rs422733	*COL4A2*	13	111,164,614	33	2714	0.92	2 × 10^–291^
rs3832425	*SSR1*	6	7,289,415	31	2281	0.92	5 × 10^–267^
rs1046138	*PKP2*	12	32,944,162	32	9156	0.84	1 × 10^–253^
rs3210020	*PDLIM1*	10	96,997,440	11	1746	0.89	4 × 10^–233^
rs1051336	*HLA-DRA*	6	32,412,592	19	10,132	0.74	8 × 10^–232^
rs9512	*ABLIM3*	5	148,639,762	30	2,580	0.22	8 × 10^–204^

The table shows the ten SNPs with the most significant ASE in the LA samples (n = 5688), the gene they are located within, the genomic location, number of heterozygous samples and reads overlapping the SNP available for ASE calling, the REF/ALT + REF ratio, and the FDR-adjusted *p* value for ASE

In the LV samples, there were 13,009 SNPs with significant ASE at *p* < 0.05 (Additional file 3). Of those, 3774 had an FDR-adjusted *p* value < 0.05 (Fig. 1b). Table 2 shows the ten most significant SNPs and Fig. 1b shows the genomic distribution of all SNPs with ASE in the LV. From 45,496 SNPs available for analysis in our LV samples and the genome-wide LV samples from GTEx, 1358 had evidence of ASE in both sample sets (*p* < 0.0001 by permutation, Additional file 1: Figure S8). Similarly, of 24,830 SNPs available for analysis in our LV samples and the exome-sequencing LV samples, 531 had evidence of ASE in both sample sets (*p* < 0.0001 by permutation, Additional file 1: Figure S9).

**Table 2:** SNPs with allele-specific expression in the LV

SNP	Gene	Chr	Location	Samples	Reads	REF/ALT + REF	FDR adj. *p* value
rs76148815	*MTR*	1	237,063,719	92	8029	0.07	5 × 10^–217^
rs7772210	*MTCH1*	6	36,936,771	58	8199	0.91	1 × 10^–209^
rs2260914	*ZSCAN18*	19	58,598,784	50	2967	0.07	2 × 10^–199^
rs1046138	*PKP2*	12	32,944,162	64	21,767	0.92	8 × 10^–194^
rs2075846	*OXA1L*	14	23,239,512	66	14,818	0.92	5 × 10^–178^
rs200574632	*MUL1*	1	20,826,784	64	3728	0.94	3 × 10^–127^
rs17850531	*ECHDC3*	10	11,805,339	56	2467	0.97	5 × 10^–109^
rs164577	*SLC30A5*	5	68,417,755	84	1374	0.03	8 × 10^–107^
rs312185	*AP2S1*	19	47,342,867	76	2040	0.96	2 × 10^–114^
rs2586306	*ABR*	17	909,451	76	2626	0.06	4 × 10^–114^

The table shows the ten SNPs with the most significant ASE in the LV samples (n = 3774), the gene they are located within, the genomic location, number of heterozygous samples and reads overlapping the SNP available for ASE calling, the REF/ALT + REF ratio, and the FDR-adjusted *p* value for ASE

### SNPs and genes with ASE in both LA and LV and differential ASE in LA compared to LV

**Fig. 2:**
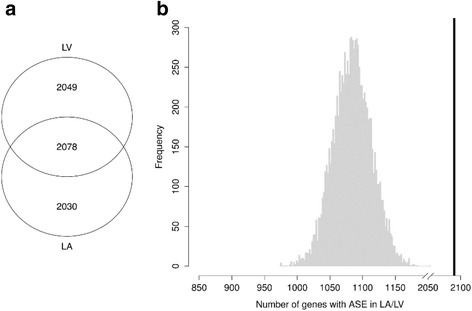
**a** A *Venn diagram* showing the number of SNPs with ASE (called at *p* < 0.05) in both LA and LV and those with ASE in one tissue but not the other. **b** The number of SNPs with ASE in both LA and LV (*black bar*, 2078) is greater than the distribution of 10,000 random draws from the SNPs available for ASE analysis (*gray* histogram)

A total of 79,538 SNPs were available for ASE calling in both the LA and LV samples and 6157 had evidence of ASE (at FDR-adjusted *p* < 0.05) in at least one tissue (Fig. 2a). Of those, 2078 had evidence of ASE in both LA and LV, a significantly increased number compared to random permutations of eligible SNPs (*p* < 0.0001 Fig. 2b). Out of the SNPs with ASE in either tissue, a significantly higher ratio of SNPs had the same direction of REF/ALT + REF ratio (either >0.5 or <0.5 in both tissue types) in the group of SNPs with ASE in both LA and LV, compared to both the group with ASE in LA and not LV (88% versus 72%, *p* < 0.0001) and the group with ASE in LV and not LA (88% versus 81%, *p* < 0.0001). Table 3 shows the top ten SNPs with ASE in both LA and LV.

**Table 3:** SNPs with allele-specific expression in both LA and LV

				LA			LV		
SNP	Gene	Chr	Location	Samples	REF/ALT + REF	FDR-adj. *p* value	Samples	REF/ALT + REF	FDR-adj. *p* value
rs3810232	*RPS9*	19	54,704,760	35	0.05	<10^-300^	76	0.03	4 × 10^–104^
rs1046138	*PKP2*	12	32,944,162	32	0.84	1 × 10^–253^	64	0.92	8 × 10^–194^
rs422733	*COL4A2*	13	111,164,614	33	0.92	2 × 10^–291^	74	0.92	1 × 10^–84^
rs202118461	*LYRM5*	12	25,357,576	32	0.86	<10^–300^	60	0.95	9 × 10^–76^
rs1060391	*YWHAB*	20	43,536,807	28	0.80	8 × 10^–164^	64	0.90	1 × 10^–99^
rs2075846	*OXA1L*	14	23,239,512	33	0.91	2 × 10^–90^	66	0.92	5 × 10^–178^
rs1042917	*COL6A2*	21	47,545,768	31	0.75	2 × 10^–161^	78	0.73	2 × 10^–72^
rs7988661	*LMO7*	13	76,427,347	11	0.72	1 × 10^–72^	30	0.74	2 × 10^–70^
rs7772210	*MTCH1*	6	36,936,771	29	0.92	9 × 10^–44^	58	0.91	1 × 10^–209^
rs7988661	*LMO7*	13	76,427,347	11	0.72	1 × 10^–72^	30	0.74	2 × 10^–70^

The table shows ten of the SNPs with significant ASE in both LA and LV, sorted by the sum of ranked *p* values for ASE calling for both LA and LV. Shown is the SNP rsID, the gene it is located within, the genomic location, number of heterozygous samples for both LA and LV available for ASE calling, the REF/ALT + REF ratio, and the FDR-adjusted *p* value for ASE for LA and LV tissues

We also tested for differential ASE in the LA compared to the baseline LV samples. This identified a total of 215 SNPs with a significantly different ASE between the two chambers at FDR *p* value of < 0.05. Table 4 shows the top ten SNPs with differential ASE in the LA compared to the baseline LV sample.

**Table 4:** SNPs with differential allele-specific expression in LA compared to LV

				LA			LV			Differential ASE LA vs. LV
SNP	Gene	Chr	Location	Samples	REF/ALT + REF	FDR-adj. *p* value	Samples	REF/ALT + REF	FDR-adj. *p* value	FDR-adj. *p* value
rs137831	*ACO2*	22	41,903,813	43	0.96	4 × 10^–99^	24	0.47	0.79	1 × 10^–75^
rs6816298	*TECRL*	4	65,144,051	17	0.39	0.001	25	0.68	4 × 10^–7^	1 × 10^–29^
rs1128416	*PPP1CB*	2	29,001,691	30	0.48	3 × 10^–5^	31	0.32	1 × 10^–34^	2 × 10^–26^
rs1046138	*PKP2*	12	32,944,162	32	0.84	1 × 10^–253^	32	0.92	8 × 10^–194^	8 × 10^–18^
rs7292	*MB*	22	36,007,045	36	0.56	3 × 10^–123^	35	0.51	1	1 × 10^–16^
rs2531332	*RPL3L*	16	1,994,749	33	0.62	2 × 10^–39^	38	0.51	0.97	3 × 10^–15^
rs201567919	*ADAR*	1	154,556,764	26	0.72	2 × 10^–52^	38	0.59	1 × 10^–8^	2 × 10^–13^
rs1063281	*TNS1*	2	218,668,732	46	0.53	0.13	39	0.46	0.003	5 × 10^–12^
rs7508	*ASAH1*	8	17,913,970	21	0.72	1 × 10^–83^	38	0.56	0.03	1 × 10^–11^
rs61756583	*OGDH*	7	44,747,514	4	0.65	2 × 10^–10^	6	0.80	2 × 10^–68^	3 × 10^–9^

The table shows the ten SNPs with the most significantly different ASE in the LA compared to LV samples (baseline pre-ischemia). Shown is the gene the SNP is located within, the genomic location, number of heterozygous samples for both LA and LV, the REF/ALT + REF ratio, the *p* value for ASE calling, and finally the FDR-adjusted *p* value for the test of differential ASE between LA and LV tissues

### Genes with differential ASE in patients with AF and ischemia

Finally, we assessed the utility of using ASE to discover novel genetic variants associated with clinical phenotypes by testing for differential allelic expression within groups of patients or paired samples. From the LA samples, the expression of each allele of available heterozygous SNPs was compared between the subgroup of patients who had poAF (34%) and those who did not. This identified 24 SNPs with differential expression of the REF and ALT alleles between the patients who had poAF compared to those who did not, at an FDR-adjusted *p* value of < 0.05 (Table 5). Of those, three SNPs had more than one heterozygous individual in both the poAF and control groups, minimizing the effects of a potential genotyping error on ASE calling. The SNP that had the most difference in ASE between the patients with poAF compared to those without was within the *GSN* gene. Two SNPs with significantly different ASE and more than one heterozygous individual in both groups, were within the *TNS1*, *LITAF*, and *CLDN18* genes (Table 5). Analysis of the functional category of the genes with differential ASE in poAF revealed enrichment within categories involved in cardiac structure, such as cardiac muscle tissue development, the sarcomere, and the myofibril (Table 6, Additional file 1: Figure S10).

**Table 5:** SNPs with differential allele-specific expression in the LA for patients with poAF

					poAF		No poAF		
SNP	Gene	Chr	Location	Reads	Samples	REF/ALT + REF	Samples	REF/ALT + REF	FDR-adjusted *p* value
rs2230287	*GSN*	9	124,065,224	10,279	1	1.00	2	0.50	1 × 10^–67^
rs41267649	*CUTA*	6	33,384,473	576	1	1.00	2	0.48	4 × 10^–40^
rs3733570	*QDPR*	4	17,503,433	538	3	0.46	1	1.00	4 × 10^–28^
rs114068468	*HLA-DPB1*	6	33,054,463	572	2	0.55	1	1.00	6 × 10^–26^
rs2241198	*IGFBP5*	2	217,539,068	642	1	1.00	3	0.52	2 × 10^–25^
rs11550240	*COX8A*	11	63,743,756	4318	1	0.56	1	1.00	2 × 10^–20^
rs1800215	*COL1A1*	17	48,265,495	688	2	0.57	1	0.03	1 × 10^–19^
rs200375051	*C7*	5	40,972,534	1081	1	0.51	3	1.00	8 × 10^–19^
rs3762568	*FASTKD2*	2	207,631,461	518	1	1.00	6	0.51	5 × 10^–18^
rs45555133	*DACT3*	19	47,151,717	288	3	0.49	2	1.00	9 × 10^–17^
rs117770959	*ZMIZ2*	7	44,807,772	207	3	0.48	1	1.00	1 × 10^–8^
rs118077107	*NEURL4*	17	7,221,197	176	1	1.00	4	0.46	5 × 10^–8^
rs74450883	*FAM219B*	15	75,193,432	361	2	0.48	1	1.00	9 × 10^–7^
rs56173620	*LAMA2*	6	129,722,453	460	2	0.51	1	1.00	2 × 10^–6^
rs11538698	*MGLL*	3	127,540,635	662	1	0.49	4	1.00	4 × 10^–5^
rs79181968	*SERPINA3*	14	95,080,814	254	1	1.00	3	0.60	9 × 10^–5^
rs12117552	*LMNA*	1	156,104,292	886	1	0.50	1	1.00	0.002
kgp10133162	*CLDN18*	3	137,750,586	188	3	0.11	5	0.60	0.004
rs138684608	*TMED10*	14	75,643,107	693	1	0.46	1	1.00	0.005
rs147227072	*PCOLCE2*	3	142,567,193	685	1	0.53	1	1.00	0.007
rs61741262	*TNS1*	2	218,669,225	3070	3	0.43	9	0.52	0.01
rs574774	*ASAH1*	8	17,914,883	3591	1	0.68	2	0.76	0.02
rs4280262	*LITAF*	16	11,647,492	1801	7	0.45	10	0.57	0.04

The table shows the SNPs with different ASE in the LA in patients who had poAF compared with those who did not. Shown is the gene the SNP is located within, the genomic location, number of reads overlapping the SNP, number of heterozygous LA samples for both patient groups, the REF/ALT + REF ratio, and the FDR-adjusted *p* value for differential ASE between the two patient groups

**Table 6:** Functional enrichment analysis of genes with differential allele-specific expression in poAF

GO annotation	FDR *p* value	Coverage
Cardiac muscle tissue development	0.008	5/85
Sarcomere	0.008	5/86
Contractile fiber part	0.009	5/102
Myofibril	0.009	5/102
Cardiac ventricle morphogenesis	0.009	4/47
Contractile fiber	0.009	5/112
Myofilament	0.012	3/16
Cardiac ventricle development	0.012	4/56
Cardiac chamber morphogenesis	0.014	4/60
Muscle structure development	0.017	6/244
Striated muscle tissue development	0.017	5/142
Cardiac chamber development	0.018	4/70
Muscle tissue development	0.018	5/150
Actin cytoskeleton	0.025	6/277
Ventricular cardiac muscle tissue morphogenesis	0.030	3/29
Ventricular cardiac muscle tissue development	0.030	3/29
Muscle organ development	0.030	5/172
Regulation of ATPase activity	0.035	3/31

The table shows the results of functional enrichment analysis of the genes with differential ASE in poAF by the GeneMANIA algorithm

From the LV samples, differential expression of reference and alternative alleles was compared between the post-ischemia sample and baseline sample in a paired manner, searching for changes in ASE as a response to ischemia. This identified three SNPs with differential expression of reference and alternative alleles between post-ischemia and baseline sample at an FDR-adjusted *p* value of < 0.05 (Table 7). The SNP with the most significant change in ASE after ischemia was within the *ZHX2* gene, followed by *CUL4A* (Table 7). Analysis of the functional category of the genes with differential ASE between the baseline and post-ischemic sample revealed enrichment within categories involved in the ubiquitin ligase complex and the regulation of transcription in response to stress (Table 8, Additional file 1: Figure S11).

**Table 7:** SNPs with differential allele-specific expression in cardiac ischemia

						Pre-bypass	Post-bypass	
SNP	Gene	Chr	Location	Reads	Samples	REF/ALT + REF	REF/ALT + REF	FDR-adjusted *p* value
rs4871331	*ZHX2*	8	123,963,996	224	2	0.35	0.64	2 × 10^–5^
kgp9659493	*CUL4A*	13	113,918,770	1541	5	0.56	0.44	0.015

The table shows the SNPs with differential ASE in the post-bypass sample compared with the baseline sample of the same individual, assessing the ASE response to cardiac ischemia. Shown is the gene each SNP is located within, the genomic location, number of reads overlapping the SNP, number of heterozygous individuals (each providing pre- and post-bypass sample), the REF/ALT + REF ratio, and the FDR-adjusted *p* value for differential ASE between the post-bypass compared to the pre-bypass

**Table 8:** Functional enrichment analysis of genes with differential allele-specific expression in cardiac ischemia

GO annotation	FDR *p* value	Coverage
Ubiquitin ligase complex	9 × 10^–14^	11/158
Cullin-RING ubiquitin ligase complex	9 × 10^–14^	10/96
Cul4-RING ubiquitin ligase complex	5 × 10^–10^	6/21
Regulation of transcription from RNA polymerase II promoter in response to stress	0.040	3/36
Protein monoubiquitination	0.040	3/36
Ubiquitin protein ligase binding	0.043	4/137
Negative regulation of cell cycle	0.043	5/291
Regulation of DNA-templated transcription in response to stress	0.043	3/41

The table shows the results of functional enrichment analysis of the genes with differential ASE in response to cardiac ischemia by the GeneMANIA algorithm

## Discussion

Here we analyzed ASE utilizing a unique dataset of high-throughput RNA-seq of a large number of samples obtained from the living human LA and LV. This eliminates artifacts induced by sampling cadaveric donors with varying degrees of warm ischemia [8]. We identified the existence of a substantial amount of ASE in both the LA and the LV of the heart. While a significant portion of the ASE is shared between the two chambers, we observed LA and LV-specific patterns of ASE. Furthermore, we show that the estimation of differential ASE can be used to identify genes potentially involved in pathogenesis of poAF and myocardial ischemia.

Quantifying global gene expression by high-throughput sequencing is traditionally done by alignment of short RNA sequence reads isolated to the tissue of interest to a previously known reference genome sequence, and subsequently to a library of known mRNA sequences and quantification of the amount of each mRNA molecule [2]. This will, however, combine the expression of both alleles, making assessment of ASE impossible. In addition to the limitations and methodological concerns involved in expression quantification, several specific issues must be considered when assessing ASE with high-throughput RNA-seq data [11]. SNP genotyping accuracy is of key importance, since incorrect genotyping of a homozygous SNP as heterozygous will lead to incorrect calling of ASE. We performed SNP genotyping using a separate assay based on DNA from peripheral blood to minimize genotyping error. A major source of bias and false positives is due to reference sequence/allele mapping bias. This is a bias introduced by the preferential alignment of RNA reads to the genome if the SNP included in the read is the reference allele, i.e. the allele included in the reference sequence used for aligning [16]. It is extremely important to quantify reference sequence bias and attempt to minimize it to reduce false positives. In this project, reference genome bias was minimized by utilizing only samples with longer read lengths and by aggressive filtering of SNPs and reads eligible for analysis. After ASE calling, some degree of reference genome bias still existed, especially for variants with fewer covering reads. Our results therefore need to be cautiously interpreted, since some SNPs with ASE are likely false positives from remaining reference genome bias. Further filtering of reads and variants to eliminate all reference genome bias is challenging, as further filtering of reads can substantially reduce the power of the analysis and other bias can be introduced by methods such as masking the reference genome [16].

Finally, the usage of simple statistical methods that are not specific to gene expression analysis to detect ASE, such as binomial test, can increase false positives and negatives since they fail to consider both inter-sample and inter-variant variability in gene expression and its measurement during differential expression calling. This was avoided by using the *edgeR* algorithm to call ASE. This utilizes the negative binomial algorithm, and estimates both the sample and marker-specific dispersion to generate variability estimates for each individual marker before calling differential expression.

After calling ASE on the filtered datasets using *edgeR*, we found that 3404 (5.1%) of SNPs eligible for ASE calling in the LA and 8642 (4.0%) of SNPs eligible for ASE calling in the LV had evidence of ASE at a FDR *p* value of <0.05. The genes with the strongest evidence of ASE in the LA were structural genes involving muscle proteins, the cardiac contraction chain (*TNNT2*, *TTN*), collagen (*COL4A2*), and cardiac energy homeostasis and cardiac fibrosis (*TIMP3*) [17, 18]. Interestingly, the SNP showing the greatest ASE in the LV sample was within the methyltransferase (*MTR*) gene involved in folate metabolism. Variants in this gene have been associated with both congenital heart disease, but only when the associated variant is inherited from the mother [19]. This suggests either global or tissue-specific imprinting of this gene, i.e. expression of each allele dependent on parental origin. Depending on the parental origin of the reference and alternative allele, this could potentially generate an ASE pattern observed within our assay, but we are limited by the lack of parental genotyping to explore this further. Also of interest is the plakophilin 2 gene (*PKP2*), associated with right ventricular cardiomyopathy [20] and multiple genes involved in mitochondrial energy metabolism (*MTCH1*, *OXA1L*, *MUL1*).

From the subset of SNPs eligible for ASE calling in both LA and LV, we found that 2078 of the SNPs had ASE in both tissues (at unadjusted *p* < 0.05), a significantly higher number than would be expected at random (Fig. 2b, *p* < 0.0001). This is in line with results from the GTEx study, where tissues with similar origin had a higher number of shared sites with ASE [8]. Especially interesting are those sites where the ASE pattern differs between LA and LV. The strongest difference was in the aconitase gene (*ACO2*), which is a part of the citric acid cycle and is essential for maintenance of mitochondrial DNA [21]. It is plausible that the control of metabolism fulfilling the differential energy needs of the two chambers is mediated via ASE.

A second aspect of our analysis was to identify SNPs/genes with differential ASE in disease states. Comparing differential ASE in the LA of individuals between those who had poAF compared to those who did not, we identified multiple interesting genes, three of which have previously been associated with AF. The gene with the most significantly different allele-specific expression was gelsolin (*GSN*), a protein that participates in cytoskeleton maintenance, modulating calcium-channels. A *GSN* mouse knockout has been found to have a short PQ interval and a prolonged QRS and QT interval, and importantly, an increased susceptibility to AF [22]. The collagen 3A1 gene (*COL1A1*) is a known target gene of the microRNA *miR29b*. The atrial expression of both *miR29b* and *COL1A1* has been found to be changed following ventricular tachypacing in a canine model, indicating that the gene might play a role in atrial remodeling following increased myocardial oxygen demand [23]. Additionally, a frameshift mutation in the lamin A/C gene (*LMNA*) has been described in a family with early-onset AF and sudden cardiac death [24]. The paucity of genes with prior association with AF in the list of genes with differential ASE in patients with poAF shows the power of utilizing ASE analysis to identify novel genes associated with cardiac disease that cannot be identified with analysis of the DNA sequence or overall mRNA expression.

Of the two genes with differential ASE and multiple individuals in both the poAF and control group, the tensin 1 (*TNS1*) gene codes for a protein with cytoskeleton contact roles and contains a motif that mediates signal transduction [25]. Variants in this gene have been associated with mitral valve prolapse [26], which was the surgical indication for a substantial number of our patients. Thus, the ASE could contribute to either AF pathogenesis or the response to mitral valve prolapse. The functional enrichment analysis of genes with differential ASE in poAF indicated an enrichment of genes involved in cardiac structure, that supports the importance of structural remodeling in the pathogenesis of AF [27].

Similarly, the comparison of ASE in paired samples before and after ischemic injury via cardioplegic arrest identified three genes of interest. The zinc finger and homeodomain protein 2 (*ZHX2*) has been identified as a modulator of plasma lipids [28] and it is plausible that regulation or dysregulation of lipid metabolism is a response to hypoxemia. Interestingly the overexpression of the ubiquitin E3 ligase gene (*CUL4A*), found to have differential ASE in our ischemia model, has been found to suppress apoptosis and DNA damage in experimental pheochromocytoma model of ischemia-reperfusion [29]. This gene has not previously been associated with response to hypoxia in cardiac cells and is an interesting target for further studies. Functional enrichment analysis highlighted the ubiquitin ligase complex pathway, but other members of this pathway, including *CHIP* and *MDM2*, have been associated with the regulation of apoptosis in ischemia-reperfusion injury [30]. It is likely that expression differences affecting this pathway are involved in the immediate response to ischemia and they serve as interesting targets for follow-up studies. Furthermore, our analysis is limited by a relatively short time and mild ischemic insult between the baseline and post-ischemic sample. More profound or prolonged ischemia might yield allele-specific expression changes within other pathways of interest or a more profound ASE-response.

## Conclusions

Our results demonstrate that patterns of tissue-specific ASE exist in the human heart, some shared between the LA and LV, as well as chamber-specific ASE. Furthermore, we have shown the utility of ASE to highlight novel genes involved in disease states. With increasing availability of RNA-seq datasets with a high number of RNA reads of sufficient length and progress in the methodology of ASE analysis, this opens novel and exciting areas to advance the understanding of the genetic background and molecular mechanisms behind the pathogenesis of common and complex diseases, including those in the human heart.

## Additional files

Additional file 1: Supplementary Table S1, Supplementary Figures S1–S11. Assessment of allele-specific expression, quality control, comparison of allele-specific expression calling algorithms, comparison of allele-specific expression between datasets, and results from functional enrichment analysis. (PDF 1091 kb)
Additional file 2: List of SNPs with significant allele-specific expression in LA. (TXT 1290 kb)
Additional file 3: List of SNPs with significant allele-specific expression in LV. (TXT 1941 kb)
