# Supplementary material for: Allele-specific expression in the human heart and its application to postoperative atrial fibrillation and myocardial ischemia
Source: Genome Med. 2016 Dec 6;8:127. doi: 10.1186/s13073-016-0381-1 (PMC5139013; doi:10.1186/s13073-016-0381-1)
Supplement: Additional file 1: — Supplementary Table S1, Supplementary Figures S1–S11. Assessment of allele-specific expression, quality control, comparison of allele-specific expression calling algorithms, comparison of allele-specific expression between datasets, and results from functional enrichment analysis. (PDF 1091 kb) [file 13073_2016_381_MOESM1_ESM.pdf]

Supplementary Table S1

	LA				LV			
	SNP's n	p<0.05 (binom)	REF %	ratio	SNP's n	p<0.05 (binom)	REF %	ratio
All SNP's chr 1-22 with 1 het pt	453,015	239,360	53%	0.54	906,116	496,791	55%	0.523
Min 15 reads	117,012	32,431	28%	0.549	222,728	77,998	35%	0.522
HW>0.00001 and genotyping>95%	116,238	32,431	28%	0.549	219,018	76,439	35%	0.521
Mappability=1	114,523	31,549	28%	0.549	215,925	75,225	35%	0.521
RM monoall/genot error	112,020	29,046	26%	0.542	214626	73,926	34%	0.520
**Final count**	**112,020**	**29,046**	**26%**	**0.542**	**214,626**	**73,926**	**34%**	**0.520**

The table shows the number of SNPs with at least 1 heterozygous patient, and the filtering by read counts, Hardy-Weinberg (HW) equilibrium and genotyping rate, estimate of mappability and removal of SNPs with potential genotyping error or monallelic expression. The table also shows the number and percentage of SNPs with a p-value for ASE by binominal test of <0.05 and the mean REF/ALT+REF ratio. LA- Left Atrium, LV – Left Ventricle.

Supplementary Figure S1

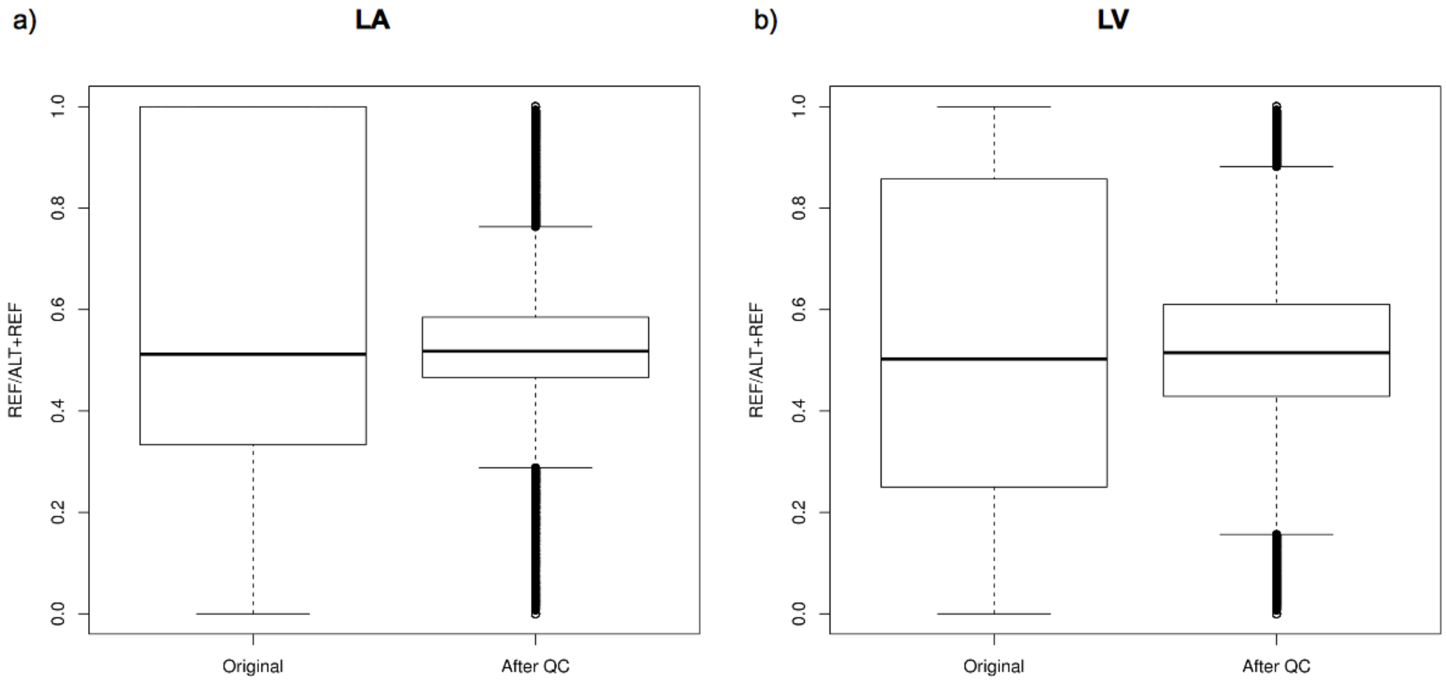

Boxplots showing the distribution of REF/ALT+REF ratios for SNPs before and after quality control (QC) Filtering of SNP, as described in text and Supplementary Table 1. Shown are results from a) left atrium (LA) and b) left ventricle (LV).

Supplementary Figure S2

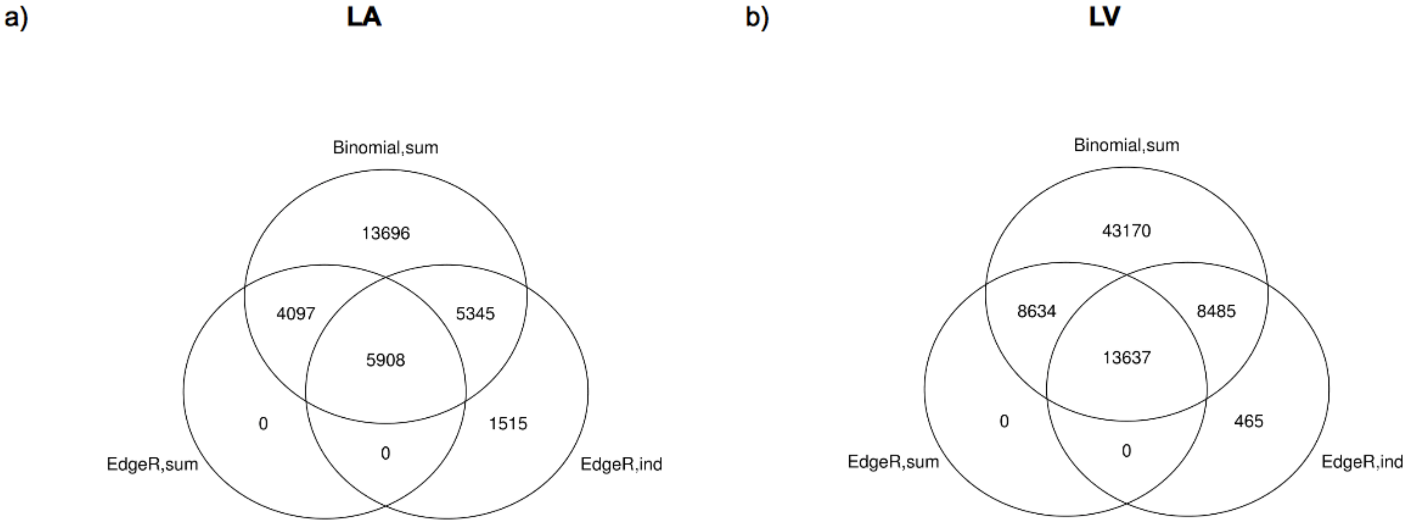

A Venn diagram showing the number of SNPs with ASE at $p<0.05$ with three algorithms tested, and their overlap. Binom, sum is the ASE called by binomial test using the sum of REF and ALT allele over all individuals, EdgeR, sum is the ASE called with the EdgeR package utilizing the negative binomial distribution with a fixed dispersion estimate of 0.1 using the sum of REF and ALT allele count over all individuals. EdgeR, ind is the ASE called using the EdgeR package utilizing allele counts from each individual. This allows for dispersion estimate using the sample results prior to ASE calling. Shown are results from a) left atrium (LA) and b) left ventricle (LV).

Supplementary Figure S3

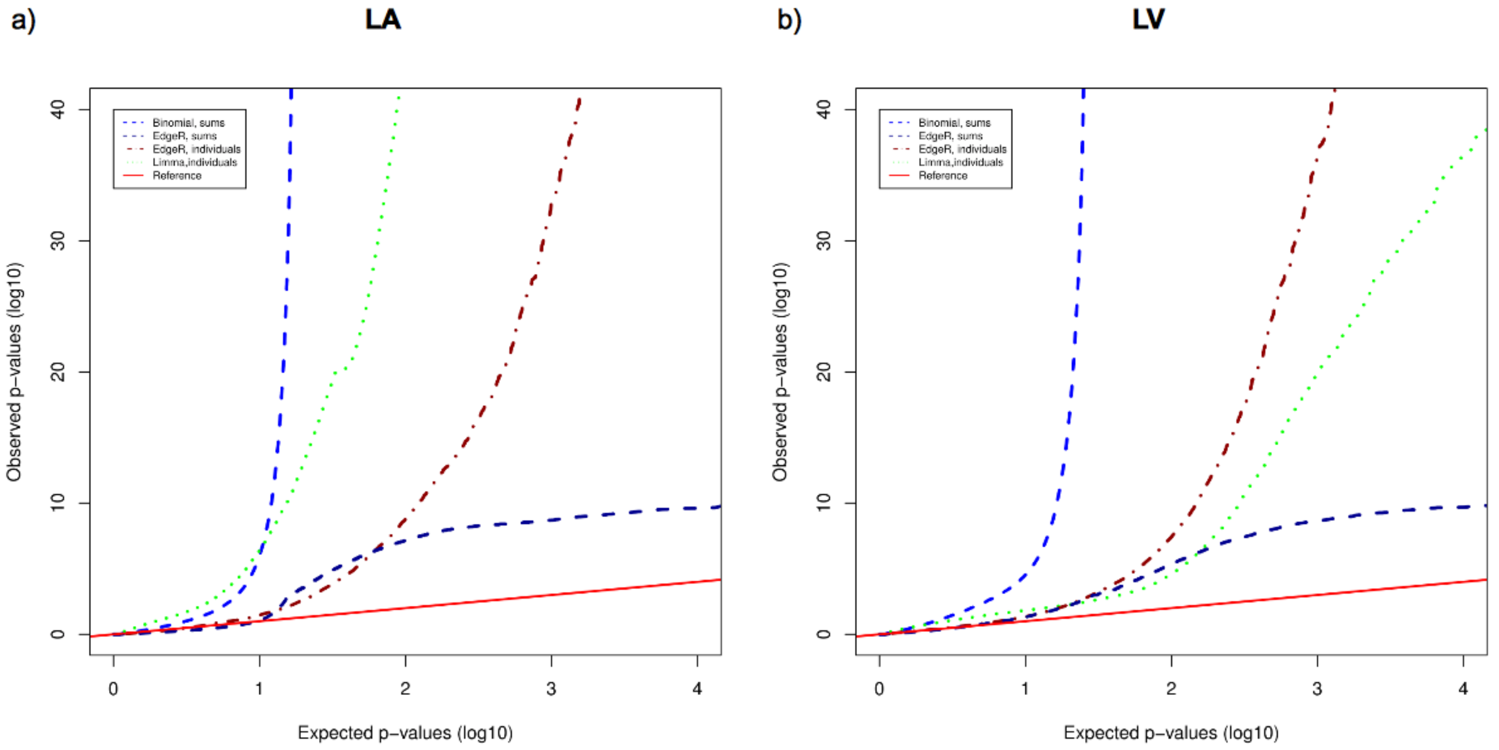

A QQ-plot of the various ASE calling algorithms tested, on a) LA or b) LV samples. This shows the observed (y-axis) against expected (x-axis) p-values for ASE calling based on binominal test from allele counts summed over all individuals (light blue), ASE calling based on edgeR negative binominal algorithm using allele counts summed over all individuals (dark blue), ASE calling based on edgeR using allele counts using individual allele counts (dark red) and ASE calling based on limma package using individual allele counts (light green), against reference (red). Shown are results from a) left atrium (LA) and b) left ventricle (LV).

Supplementary Figure S4

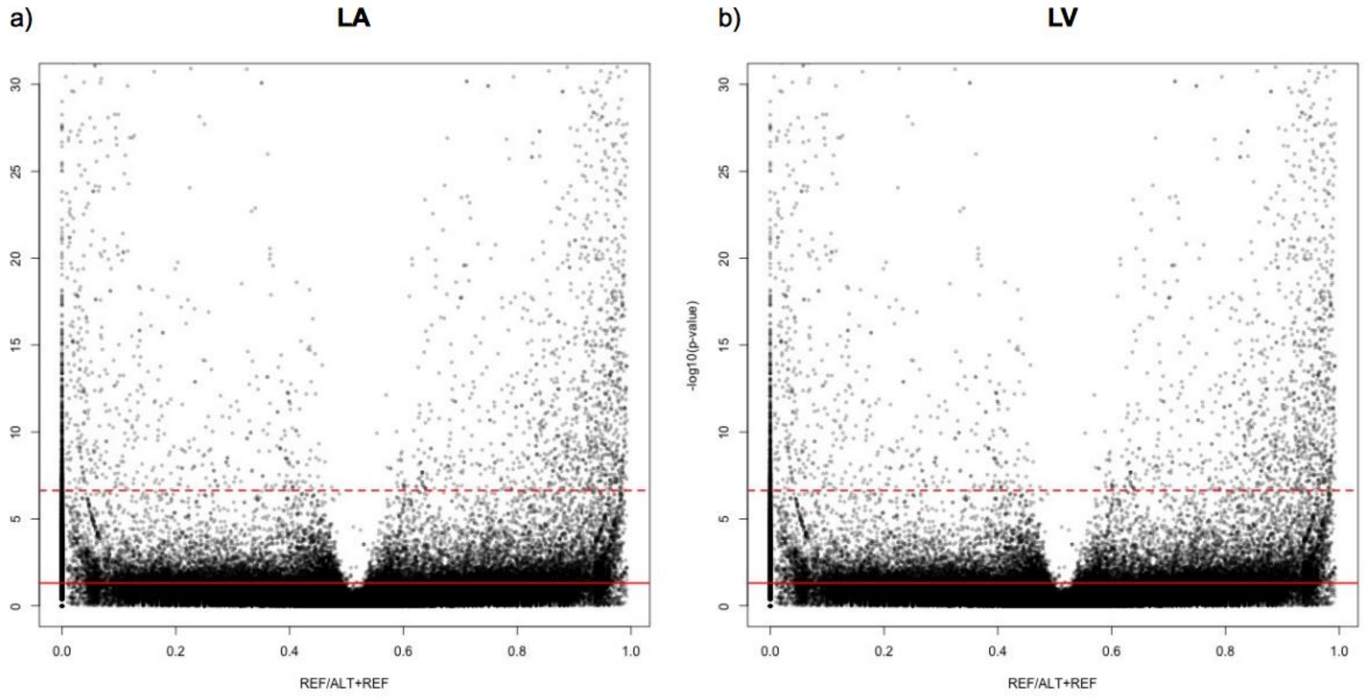

A plot of REF/ALT+REF ratio (x-axis) compared to the negative logarithm of the p-value for ASE calling, using the EdgeR algorithm utilizing individual counts of REF and ALT alleles. A REF/ALT+REF ratio of 0.5 indicates equal expression of REF and ALT alleles and no ASE. Shown are results from a) left atrium (LA) and b) left ventricle (LV). Horizontal lines indicate p=0.05 (solid red), and p=0.05/n where n is the number of SNPs tested in each tissue (broken red).

Supplementary Figure S5

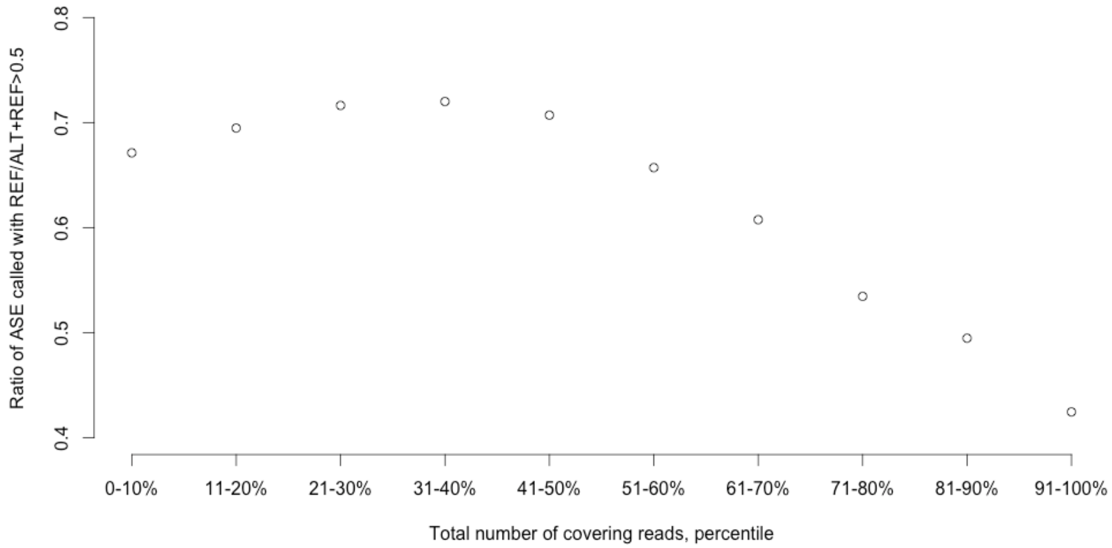

A plot of the ratio of SNPs with significant ASE called using the EdgeR algorithm with a REF/ALT+REF ratio >0.5, compared against the number of reads covering each SNP(x-axis). The SNPs were divided into ten groups based on the number of covering reads.

Supplementary Figure S6

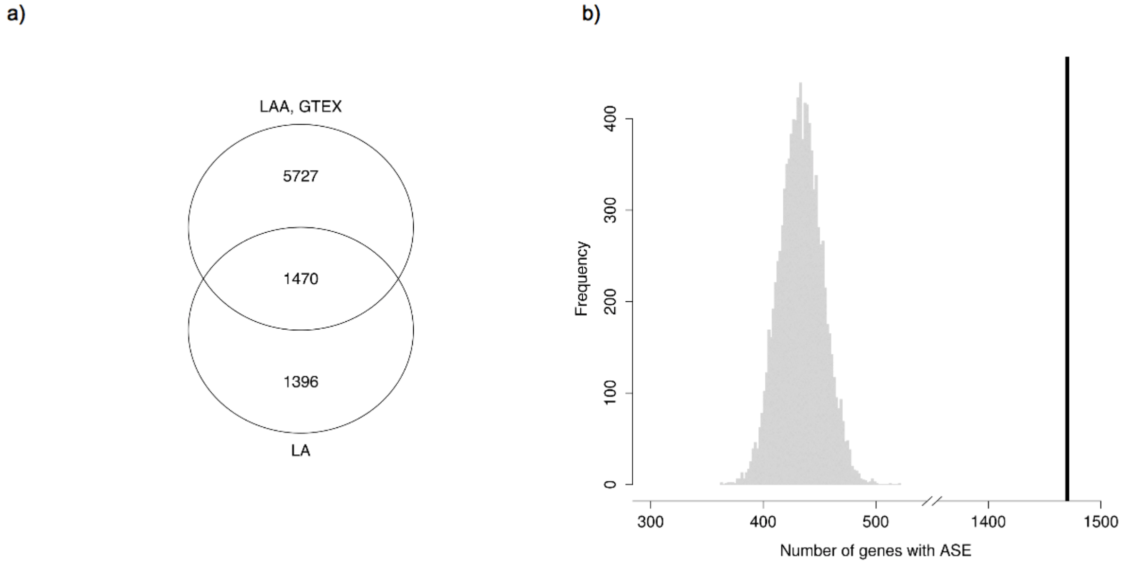

a) A Venn diagram showing the number of SNPs with ASE (called at FDR adjusted $p<0.05$) shared between our left atrium (LA) dataset and the GTEx LA appendage whole genome RNA sequencing dataset, and number of SNPs with ASE in one dataset but not the other. b) The number of SNPs with ASE in both datasets (black bar) is greater than the number of SNPs shared between two random draws of eligible SNPs (grey histogram, 10,000 permutations).

Supplementary Figure S7

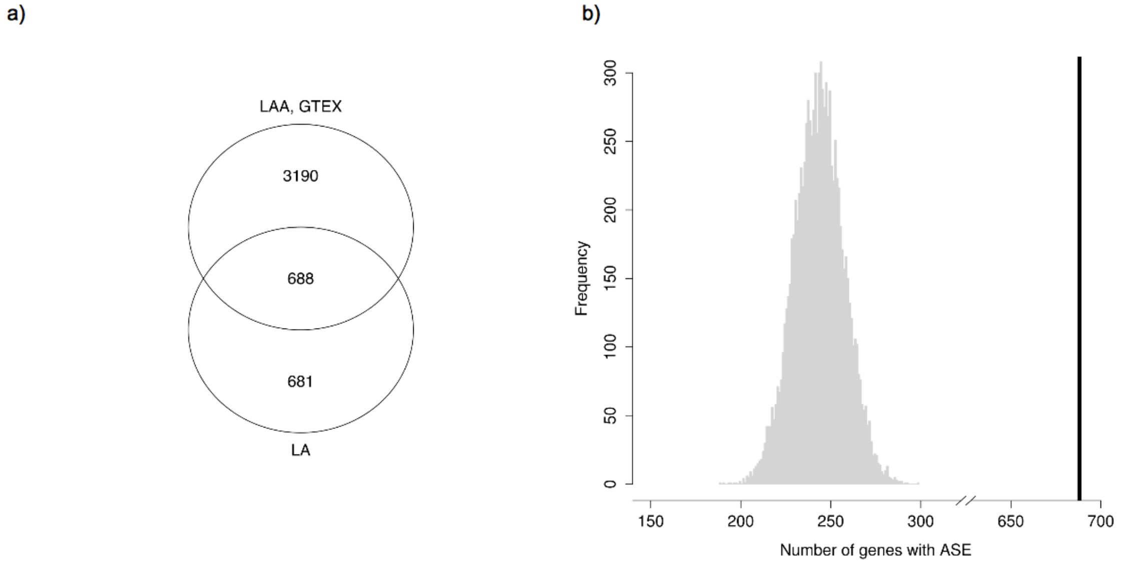

a) A Venn diagram showing the number of SNPs with ASE (called at FDR adjusted $p<0.05$) shared between our left atrium (LA) dataset and the GTEx LA appendage exome RNA sequencing dataset, and number of SNPs with ASE in one dataset but not the other. b) The number of SNPs with ASE in both datasets (black bar) is greater than the number of SNPs shared between two random draws of eligible SNPs (grey histogram, 10,000 permutations).

Supplementary Figure S8

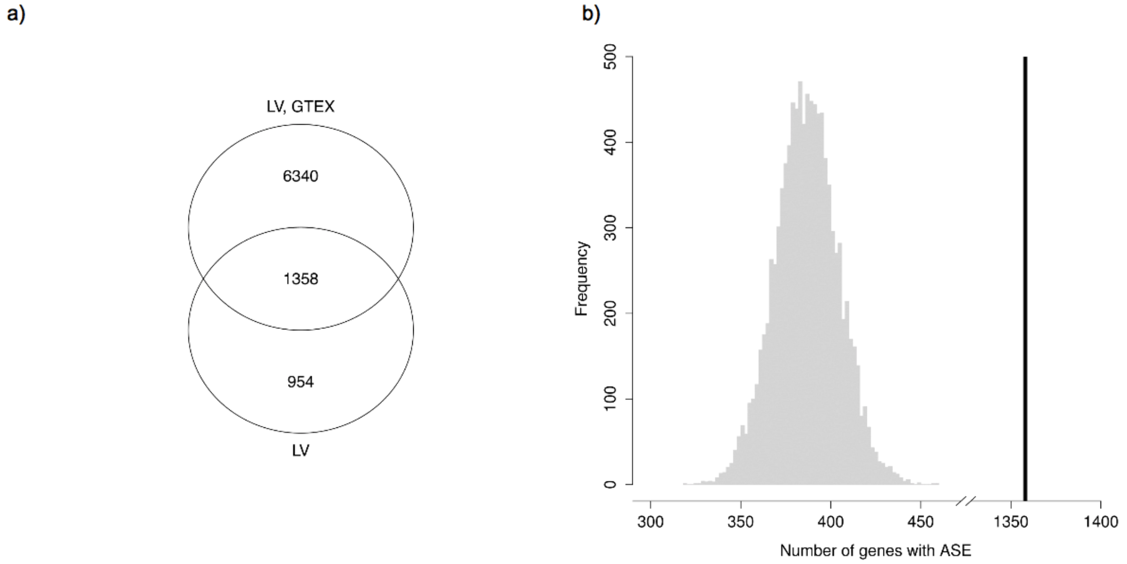

a) A Venn diagram showing the number of SNPs with ASE (called at FDR adjusted *p*<0.05) shared between our left ventricle (LV) dataset and the GTEx LV whole genome RNA sequencing dataset, and number of SNPs with ASE in one dataset but not the other. b) The number of SNPs with ASE in both datasets (black bar) is greater than the number of SNPs shared between two random draws of eligible SNPs (grey histogram, 10,000 permutations).

Supplementary Figure S9

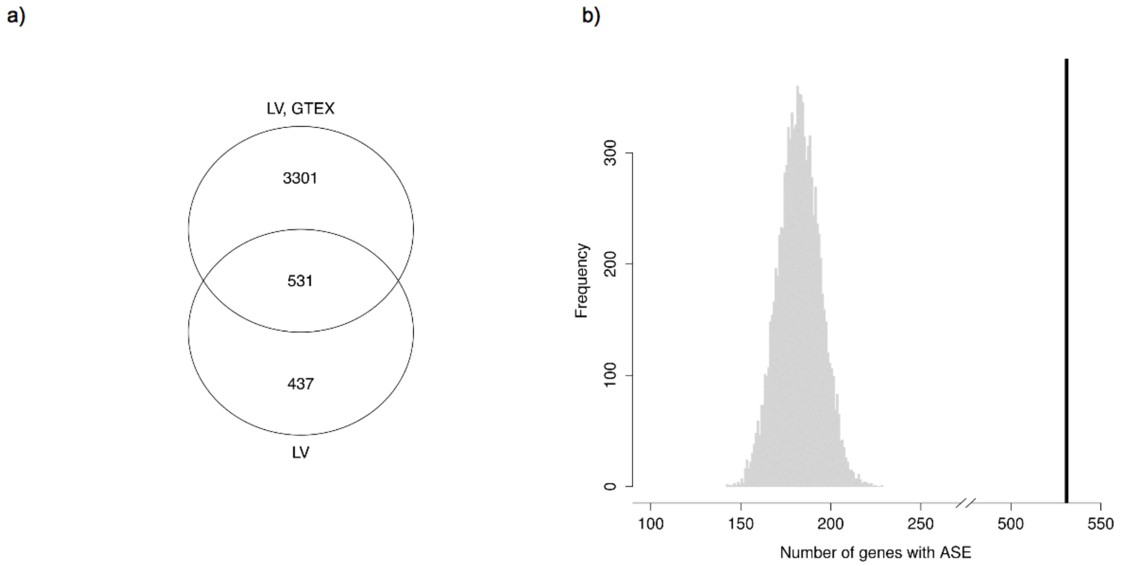

a) A Venn diagram showing the number of SNPs with ASE (called at FDR adjusted $p<0.05$) shared between our left ventricle (LV) dataset and the GTEx LV exome RNA sequencing dataset, and number of SNPs with ASE in one dataset but not the other. b) The number of SNPs with ASE in both datasets (black bar) is greater than the number of SNPs shared between two random draws of eligible SNPs (grey histogram, 10,000 permutations).

Supplementary Figure S10

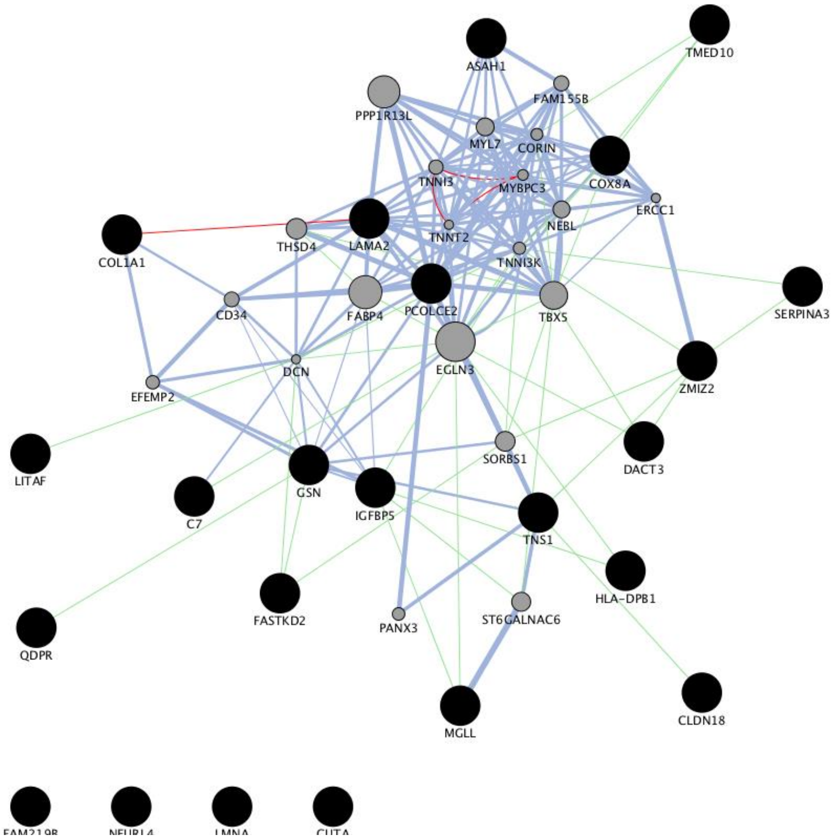

Results of the functional network analysis of the list of genes with differential allele-specific expression in the left atrium (LA) of patients with post-operative atrial fibrillation (poAF) in by the GeneMANIA algorithm. The algorithm was set to run on default networks, with substitution of the default co-expression networks for custom-made LA co-expression network. In addition to genes with differential ASE (black), the algorithm allowed the inclusion of the top 20 related genes and at most 20 attributes using automatic weighting (gray). Connections were established in the context of co-localization (blue connections), genetic interactions (green) or known pathways (red) networks.

Supplementary Figure S11

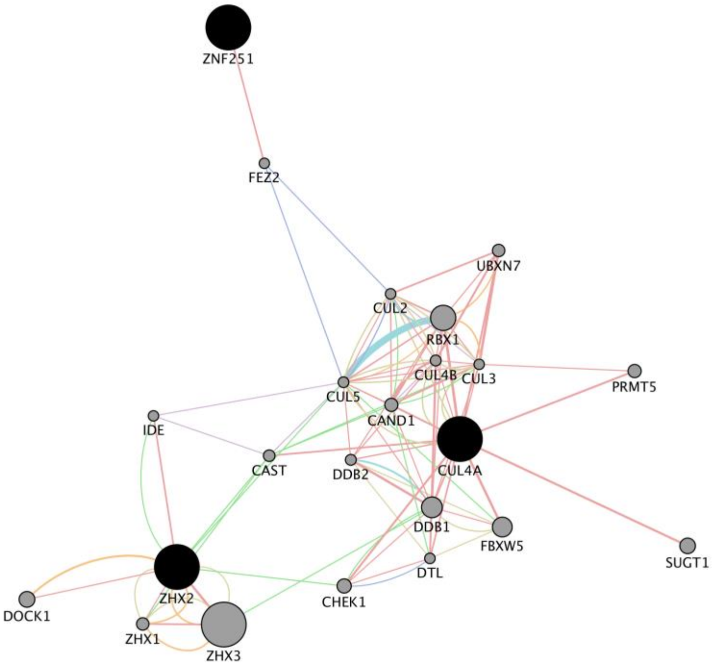

Results of the functional network analysis of the list of genes with differential allele-specific expression in the left ventricle (LV) after cardiac ischemia by the GeneMANIA algorithm. The algorithm was set to run on default networks, with substitution of the default co-expression networks for custom-made LV co-expression network. In addition to genes with differential ASE (black), the algorithm allowed the inclusion of the top 20 related genes and at most 20 attributes using automatic weighting (gray).

Connections were established in the context of physical interactions (red connections), predicted pathways (orange), known pathways (light blue), co-localization (dark blue), genetic interactions (green), shared protein domains (brown) and co-expression (gray) networks.
